# Line drawings as a tool to probe edge sensitivity in natural scenes

**DOI:** 10.1167/jov.25.14.22

**Published:** 2025-12-23

**Authors:** Lynn Schmittwilken, Anna L. Haverkamp, Marianne Maertens

**Affiliations:** 1 Computational Psychology, Electrical Engineering and Computer Science Technische Universität Berlin, Berlin, Germany

**Keywords:** edge sensitivity, pattern vision, object segmentation, natural scenes, psychophysics

## Abstract

To interact with the world effectively, the human visual system must extract meaningful features from visual scenes. One key feature are edges, luminance or texture discontinuities in two-dimensional (2D) images that often correspond to object boundaries in three-dimensional scenes. Edge sensitivity has traditionally been studied with well-controlled stimuli and binary choice tasks, but it is unclear how well these insights transfer to real-world behavior. Recent studies have extended this approach using natural images but typically retained binary button presses. In this study, we extend the approach further and ask observers (*N* = 20) to trace edges in natural scenes, presented with or without 2D visual noise. To quantify edge detection performance, we use a signal detection theory–inspired approach. Participants' edge traces in the noise-free condition serve as an individualized “ground-truth” or signal, used to categorize edge traces from noise conditions into hits, false alarms, misses, and correct rejections. Observers produce remarkably consistent edge traces across conditions. Noise interference patterns mirror results from traditional edge sensitivity studies, especially for edges with spectral properties similar to natural scenes. This suggests that insights from controlled paradigms can transfer to naturalistic ones. We also examined edge traces to identify which image features drive edge perception, using interindividual variability as a pointer to relevant features. We conclude that line drawings are a powerful tool to investigate edge sensitivity and potentially other aspects of visual perception, enabling nuanced exploration of real-world visual behavior with few experimental trials.

## Introduction

A central challenge in vision research is to understand how the visual system extracts meaningful features from the visual input. One such feature are edges (Marr, 1976; Georgeson, May, Freeman, & Hesse, 2007; Morgan, 2011). Edges often occur at object boundaries, and hence play a critical role in segmenting visual scenes into perceptually meaningful units (Marr & Hildreth, 1980; Bell & Sejnowski, 1997). As a result, edge detection has been a central theme in the study of vision, both in humans (Morgan, 2011; Sayim & Cavanagh, 2011) and in computer vision systems (Ferrari, Fevrier, Jurie, & Schmid, 2007; Zitnick & Dollár, 2014).

Edges can be formally defined as spatial discontinuities in luminance. Their visibility depends on the sharpness of the transition and the contrast between the two sides of the edge. Consequently, edge detection and contrast sensitivity are closely linked. Much of our understanding of these phenomena comes from traditional psychophysical experiments with well-controlled stimuli (i.e., isolated edges or low-contrast gratings) (Campbell & Robson 1968; Shapley & Tolhurst, 1973; Elder & Sachs, 2004), and binary responses (i.e., button presses that indicate whether or not a stimulus was perceived). These methods have been instrumental in advancing our understanding of vision at the limit and under controlled conditions (Swets, 1961; Rust & Movshon, 2005), but they may not fully capture the complexity of visual behaviors in natural scenes (Felsen & Dan, 2005; Olshausen & Field, 2005; Bex & Langley, 2007; Neri, 2017, Neri, 2024).

One well-established finding from traditional psychophysics is the contrast sensitivity function (CSF), which describes the relationship between sensitivity and the spatial frequency (SF) content of the visual input. However, it is not clear how the CSF relates to edge perception at high contrast or even to apparent contrast (Georgeson & Sullivan, 1975; Haun & Peli, 2013). We recently tested sensitivity to edge stimuli with high contrast in the presence of visual noise (Schmittwilken et al., 2024). We used Cornsweet edges with different SFs (0.5, 3, and 9 cpd), embedded in broadband (white, pink, brown) and narrowband noises (center SF: 0.5, 3, 9 cpd). We found that edge sensitivity peaked around 3 cpd, which is consistent with the CSF (Shapley & Tolhurst 1973; Peromaa & Laurinen, 2004). Yet, we also observed masking effects, which cannot easily be predicted by the CSF. For example, 3 cpd noise masked both low and high SF edges effectively, while low SF noises (0.5 cpd and brown noise) had little to no masking effect on any edge.

**Figure 1.**
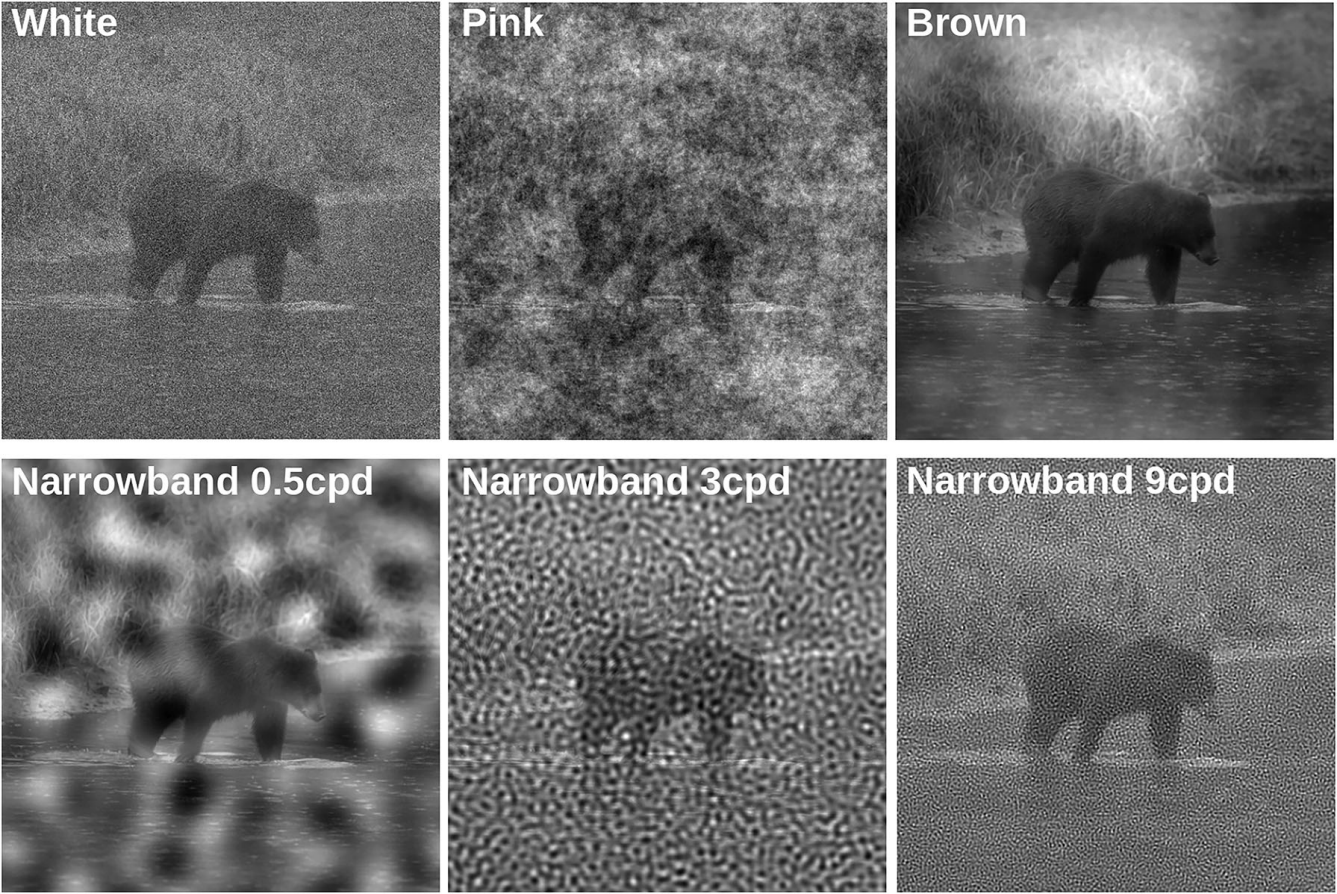
Noise conditions. Example image masked with the six types of noise used in this study. Noise contrast is constant in this demonstration.

Here we test how humans perceive edges in images of real-world scenes using a more ecologically valid task: line drawings (Sayim & Cavanagh, 2011; Fan, Bainbridge, Chamberlain, & Wammes, 2023. The images are shown at high contrast in the presence and absence of noise. We use the same noise conditions as in the previous study (Figure 1). Instead of binary detection judgments, we ask observers to actively trace the visible edges in each image with a pen. Tracing or drawing lines is a natural task for human observers and has previously been used to probe visual cognition and perceptual decision-making (Fan, Yamins, & Turk-Browne, 2018; Bainbridge, Hall, & Baker, 2019). Line drawings generate a rich dataset that provides quantitative and qualitative insights into the perceptual and cognitive organization of visual representations of the world. Below we describe our method in detail, but here is an intuition for our approach.

To quantify edge sensitivity, we compare each observer's edge traces in a noisy image (the “noisy map”) to their individual traces of the same image without noise (the “ground-truth” map). It is important to note that this “ground truth” is a subjective measure of each observer. We formulate the comparison between contours perceived in the presence and absence of noise in terms of signal detection theory. We define the ground-truth traces as *signal* and the noisy traces as *response*. This allows us to approximate true and false positives and true and false negatives, while accounting for variability in the precise placement of lines across observers and conditions.

To assess how closely the edge traces reflect traditional measures of edge sensitivity, we compared our edge sensitivity patterns from natural images to those obtained with simple edge stimuli from previous experiments (Schmittwilken et al., 2024). To anticipate, the results are broadly consistent with each other as edge sensitivity in natural images mirrors sensitivity to both 0.5 and 3 cpd edges. This is interesting because the power spectra of natural images and the simple edges are not identical. It must therefore be that shared parts of the stimulus spectra contribute to successful edge extraction in isolated edges and natural images. We also performed some exploratory image feature analysis and found that shadow edges are detected less frequently than similarly salient nonshadow edges. These examples show that our paradigm enables both (a) the kinds of analyses that are performed in traditional approaches and (b) analyses of features that drive edge perception in natural scenes looking at across-subject consistency.

## Methods

All code and the raw and processed experimental data are available at https://doi.org/10.17605/OSF.IO/Y2FDQ. These include all edge traces for each subject and condition.

### Stimuli

Visual noise is a powerful tool to study visual function (Allard, Faubert, & Pelli, 2015). One key advantage of using noise in the study of natural vision is that it allows to probe visual sensitivity to high-contrast stimuli. These are more representative of stimulus contrasts during natural viewing conditions. The choice of noise types depends on the question that is being explored.

Here, we use the same noise patterns that we recently used to probe SF selective mechanisms of human edge sensitivity with a more traditional approach (Figure 1; Schmittwilken et al., 2024). Using the same noises allows us to (a) validate our method against an existing empirical dataset and (b) compare edge sensitivity patterns between a naturalistic and a more controlled paradigm.

Natural images were taken from the Contour Image Database (Grigorescu, Petkov, & Westenberg, 2003), which contains 40 grayscale images (512×512 pixels) of scenes depicting animals in their natural habitat and some human-made objects. Each image comes with a human-drawn contour map, which can serve as a neutral comparison point independent of our experiment. An example image from the database with the six different noise patterns from Schmittwilken et al. (2024) is shown in Figure 1. Stimulus size was 11.6 degrees of visual angle. Mean luminance was 100 cd/m^2^. Our experimental design required a total of 30 images from the database. We decided to select the 30 images with most similar *perceived* contrasts, as we quantified in a pilot study.

### Task

For more than 30,000 years, humans have displayed and communicated visual information with line drawings (Fan et al., 2023, for review). This makes line drawings an interesting task to probe visual sensitivity in natural scenes. Since line drawings are more informative than simple button presses, they might provide additional clues to the visual features that guide observers' drawing behaviors, as well as their (cognitive) strategies (Bainbridge, 2022).

During the experiment, observers were shown natural images of varying contrast and with superimposed noise masks in random order (Figure 1). Subjects were instructed to trace all visible edges in the stimulus on a drawing pad that was placed in front of them. We defined edges as image regions in which a sudden luminance change occurs, which is often the case at object boundaries or their elements. We told them not to trace fine-grained textures, such as grass, and not to fill in image regions. We emphasized that observers should draw all edges visible to them but avoid drawing edges based on logical completions or continuations. Finally, we asked them to not trace the noise patterns.

### Experimental design

To compare our results to Schmittwilken et al. (2024), we quantified the effect of the six noise patterns on edge visibility, measuring one psychometric function for each noise type. Each psychometric function was sampled at five image contrasts, which were manually selected for each noise condition in prior piloting. The image contrasts were chosen to sample the full performance range in each noise condition, from barely seeing any edges in the images to seeing most of them. Noise contrasts were fixed at a root-mean-squared (RMS) contrast of 0.1 (standard deviation divided by mean luminance) to ensure that all noises had the same contrast energy (i.e., mean power).

**Figure 2.**
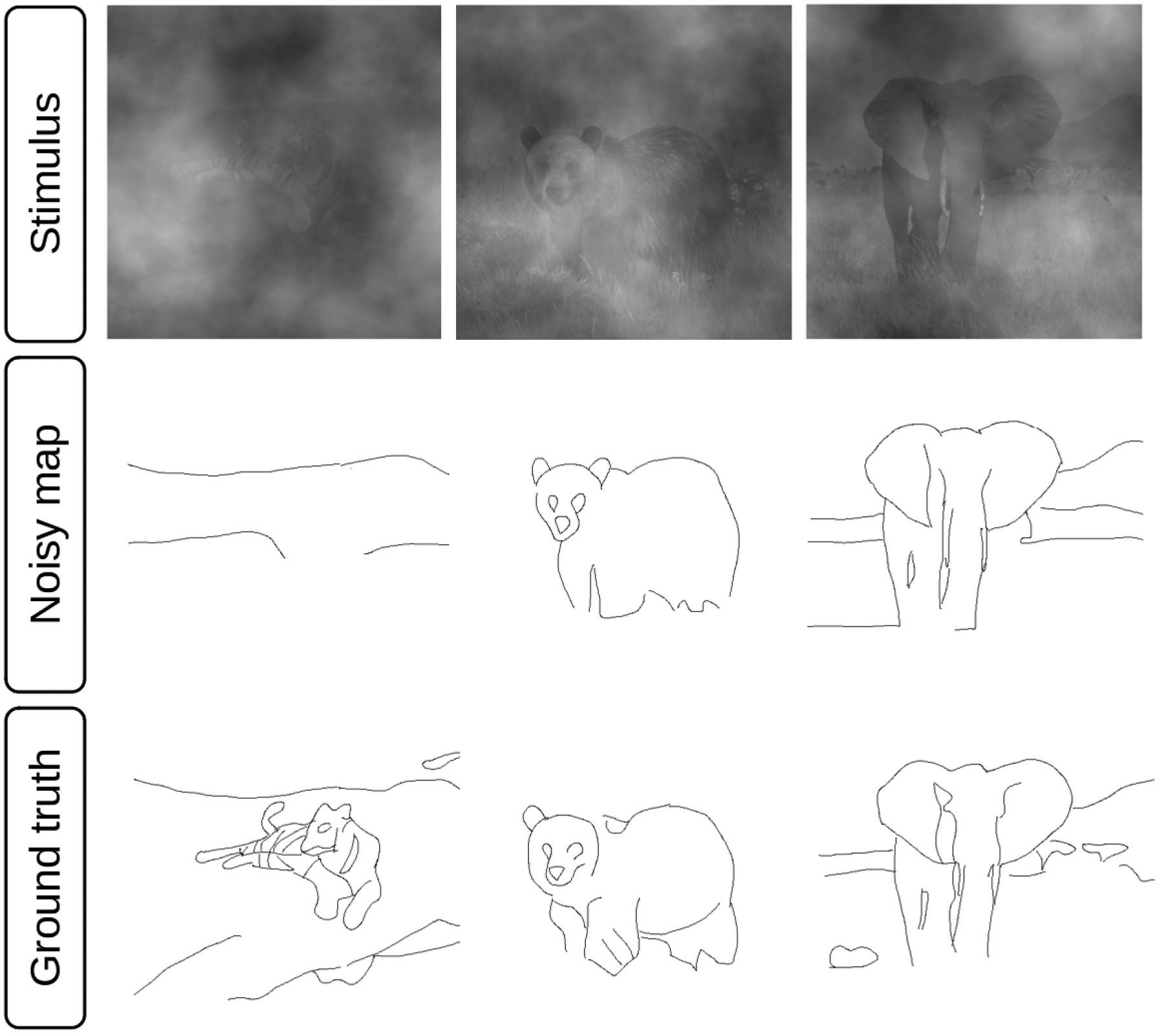
Design. Example traces (center row) drawn by one subject for the three stimuli in the top row with brown noise. Image contrast is increasing from left to right. Noise contrast is constant. The bottom row shows the traces of the same subject for the same stimuli at higher contrast and without noise. We compute performance as agreement between the ground-truth maps and the noisy maps for each subject.

The experiment was split into two sessions. In the first session, subjects were presented with the preselected 30 natural images in noise in random order. Image contrast and noise type were randomly selected from the 30 possible options (5 contrasts × 6 noises). As a result, a given combination of contrast and noise was likely used on different image identities across observers. Each subject saw each image identity exactly once to avoid memory effects. Figure 2 shows three example stimuli for one observer in the presence of brown noise (top row) and the corresponding segmented traces (central row).

In the second session, we asked observers to perform the drawing task for the 30 images once more. The images were presented at high contrast (RMS = 0.16) and in the absence of visual noise. The resulting noise-free traces (Figure 2, bottom row) provide the basis for quantifying edge visibility and provide an opportunity to investigate interindividual differences due to differences in personal biases, cognitive strategies, drawing style, or drawing skills.

### Performance measures

We use a signal detection theory (SDT) framework (Green & Swets, 1966) to quantify edge-tracing performance in natural images in the presence of noise. To apply SDT, studies typically employ simple tasks, such as single-interval detection or two-alternative forced-choice experiments (Macmillan & Creelman, 1991). In these tasks, results are categorized based on whether the signal was present or absent and whether the response was correct or incorrect. This generates four categories in a typical contingency table, from which measures for sensitivity and response biases can be derived:

• Hit: signal was present and was detected.
• Miss: signal was present but not detected.
• False alarm (FA): signal was absent but was reported as present.
• Correct rejection (CR): signal was absent and reported as absent.

**Figure 3.**
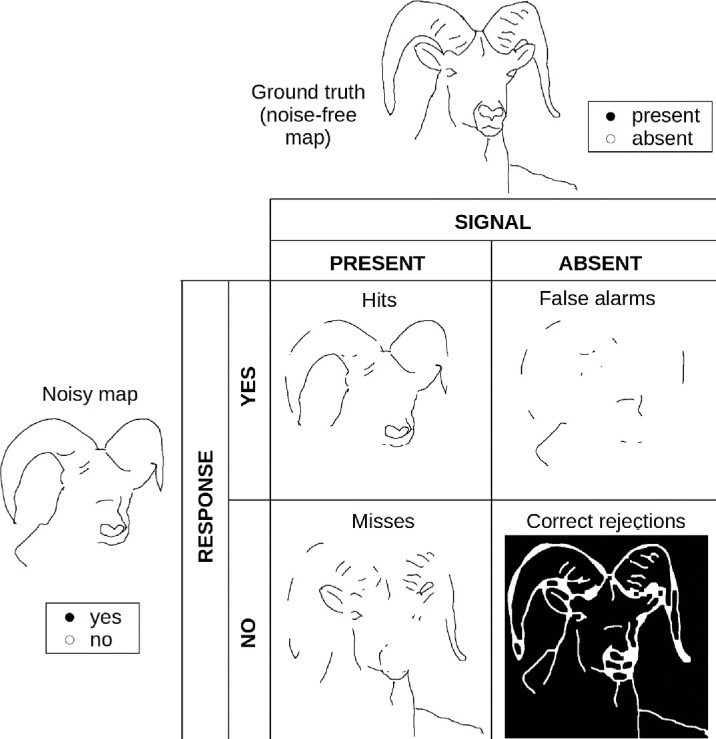
Performance evaluations. The figure shows how we apply SDT in our paradigm. We compare subjects' ground-truth maps (signal) with their noisy maps (response) and count the number of hits, misses, false alarms, and correct rejections per image. It might seem odd that some pixels are classified as false alarms, although they are part of the outline (e.g., part of the horn). This is due to drawing inaccuracies of observers. As we explain below (error margin), we used an error margin to compensate for these drawing inaccuracies, but the alignment between observers' edge maps in the presence and absence of noise is still not perfect.

Here we compare observers' line drawings in the presence and absence of noise and treat each pixel as an individual trial. Different from classical SDT tasks, we rely on the subjective experience of observers to define the “signal.” The signal was present when an observer traced the contour in the noise-free image regardless of whether or not there was a physical edge in the image. We refer to these noise-free traces as *ground truth*. The traces in the presence of visual noise represent subjects' *response* (Figure 3).

This results in the following response categories:

• Hit: a pixel was classified as an edge in the ground truth and in the noisy map.
• Miss: a pixel was traced as an edge in the ground truth but not in the noisy map.
• False alarm (FA): a pixel was not traced as an edge in the ground truth but in the noisy map.
• Correct rejection (CR): a pixel was not traced as an edge in the ground truth and not traced in the noisy map.

Finally, we derive three measures of observers' response behavior from these categories, which are variations of standard SDT measures: proportion correct, d-prime, and response bias.

To fit psychometric functions, we compute *proportion correct* (*p*) as^1^:

(1) $$p=\frac{\text{Hits}}{\text{Hits}+\text{Misses}+\text{FA}}$$

**Figure A1.**
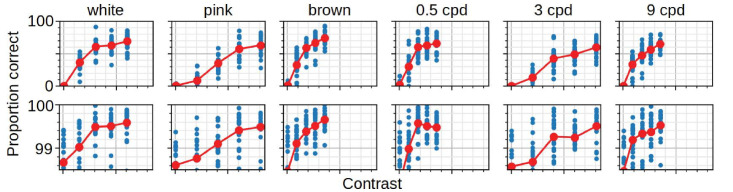
The effect of including correct rejections. The top row shows proportion correct for all noise conditions, calculated without correct rejections. The bottom row shows the respective curves for when we include the number of correct rejections to compute proportion correct. The curves barely change qualitatively, yet because of the large number of correct rejections (background pixels) in the task, they restrict the range of proportion correct from 0–100% to 99–100%. Therefore, we exclude them from analysis. Red markers represent the average across subjects; blue markers represent individual data.

Traditionally, proportion correct contains the number of correct rejections in both the nominator and the denominator. In our case, correct rejections represent pixels that were not traced and were therefore correctly identified as “non edges” or “background” (Figure 3). Since the vast majority of pixels belongs to this category, they add little information but restrict proportion correct to a narrow range. We thus leave them out with no qualitative differences (see Appendix Figure A1).

**Figure 4.**
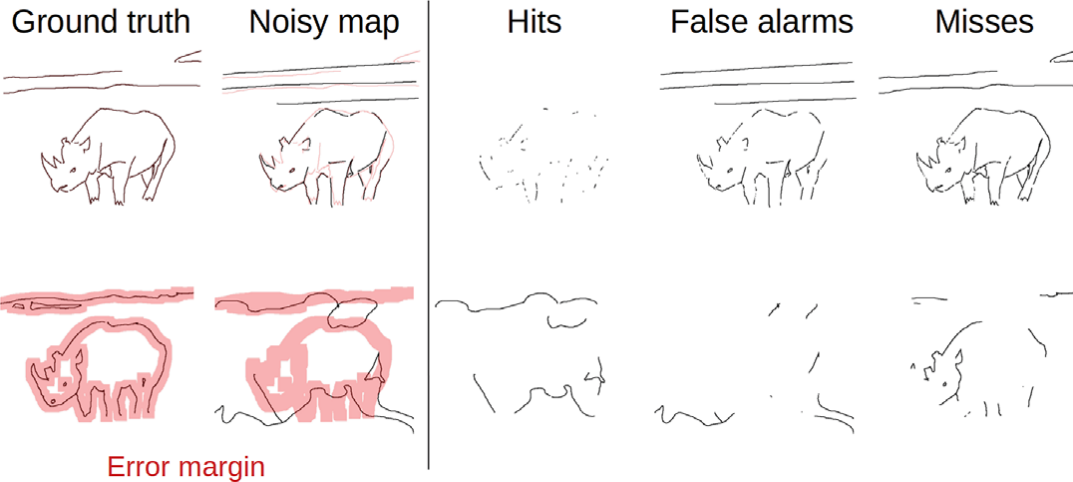
Balancing the error margin. The two examples demonstrate the impact of too small (top) and too large error margins (bottom). When the error margin is small, many valid traces are excluded as hits due to minor deviations from the ground truth. Conversely, an excessively large error margin accepts many traces as hits, even if they clearly appear as noise to a human observer.

**Figure 5.**
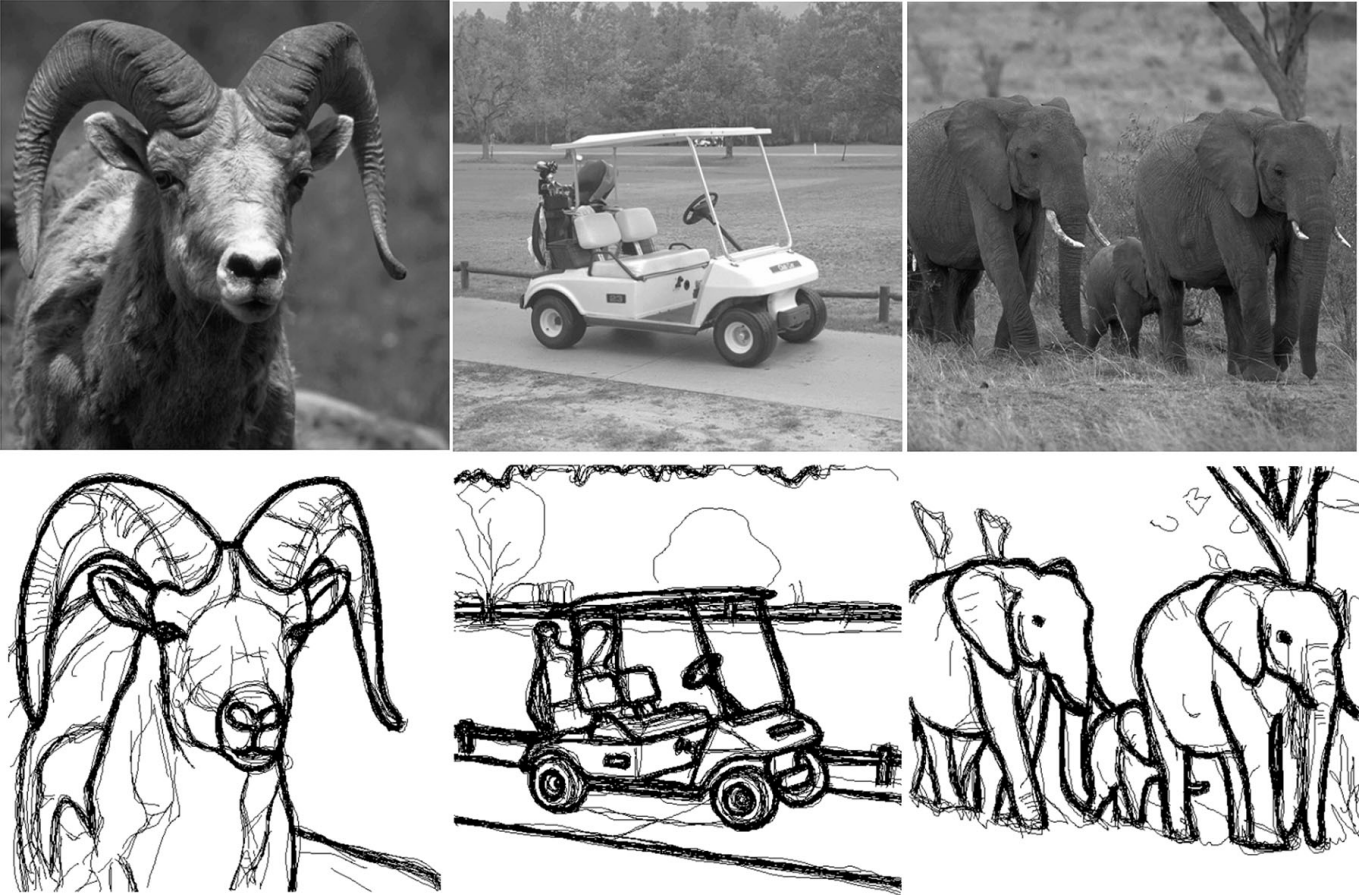
Ground-truth maps. Three example stimuli from the database (top) and the corresponding ground-truth maps of all observers overlaid on top of each other (bottom).

**Figure 6.**
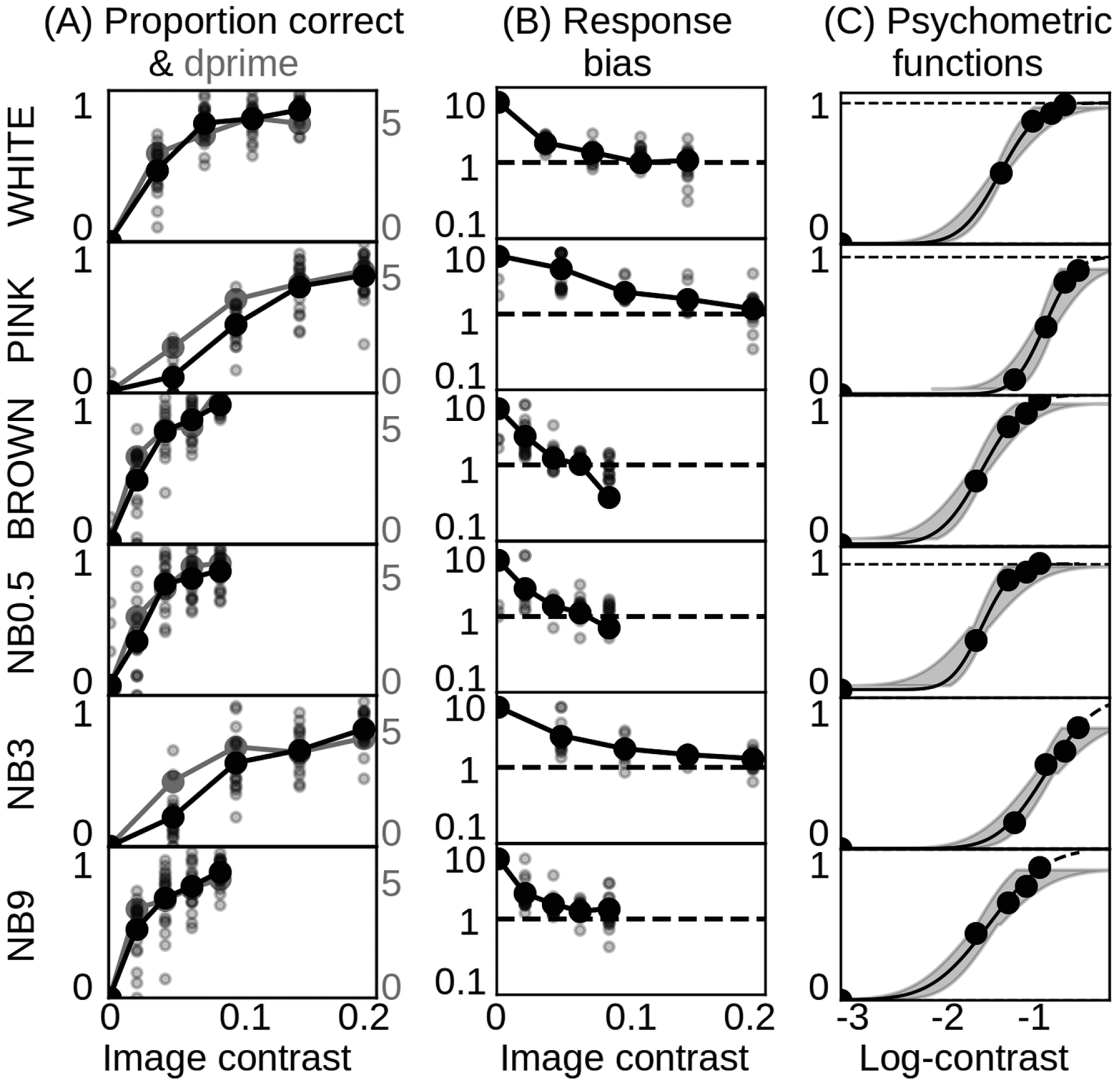
Performance curves. (A) Proportion correct and d-prime, (B) response bias, and (C) psychometric functions with increasing image contrast for all noise conditions. Response biases *c* > 1 indicate a bias toward “signal present,” while *c* < 1 indicates a bias toward “signal absent.” Large markers represent the average data across all *N* = 20 observers. Small markers represent individual data. Shaded areas indicate 68% credible intervals.

**Figure 7.**
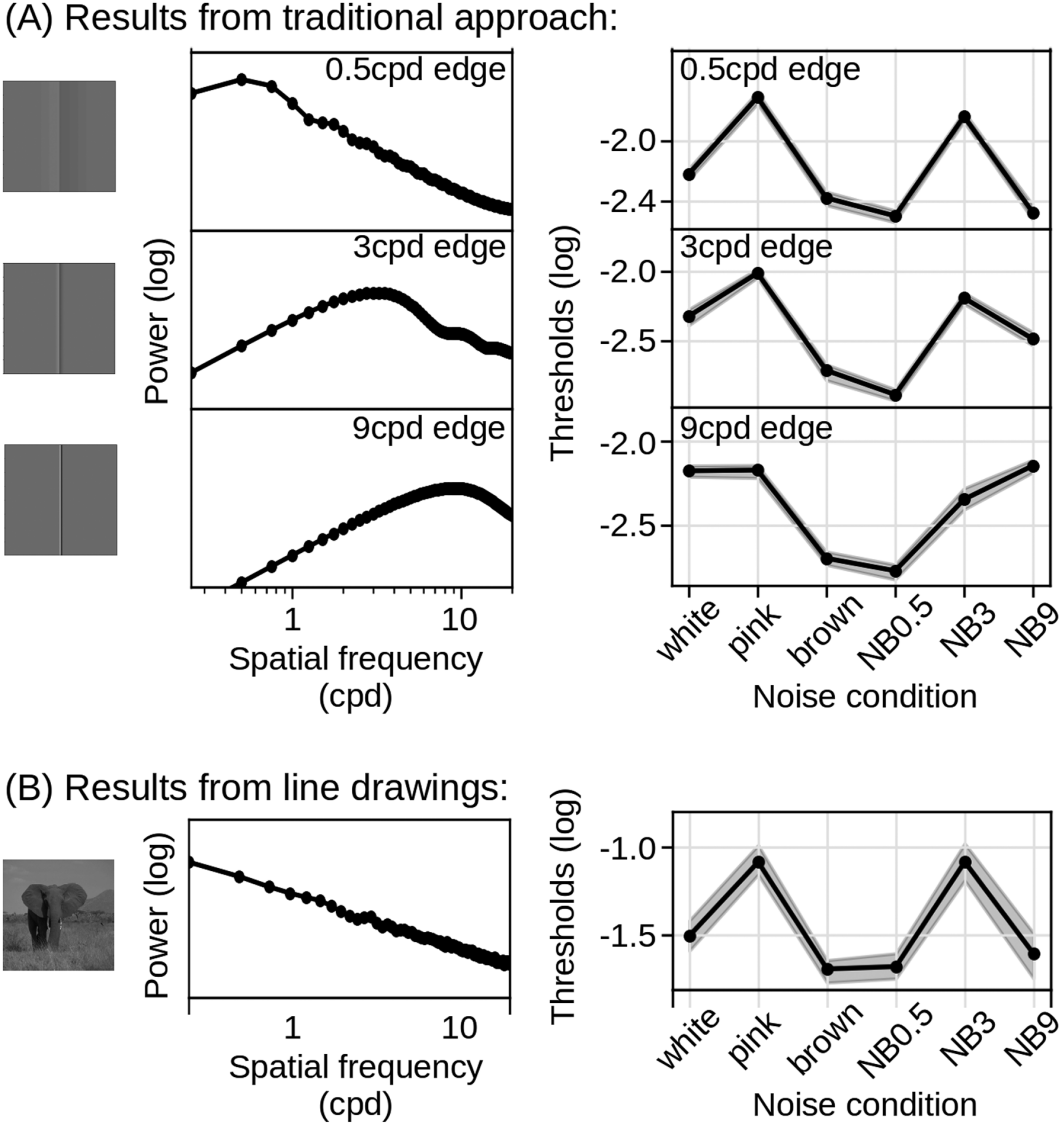
Comparison with traditional approach. (A) Summary of results from Schmittwilken et al. (2024), who probed edge sensitivity to Cornsweet edges with three peak frequencies and the same noise masks as used in the present study. The left column shows the three edge types, the central column the corresponding power spectra, and the right column detection thresholds obtained in a 2-AFC task. (B) For comparison, the same information is shown for our line drawing approach with natural images. Note that both the power spectra and the threshold patterns obtained with natural images most closely resemble those of the 0.5 cpd edge. NB: Narrowband.

**Figure 8.**
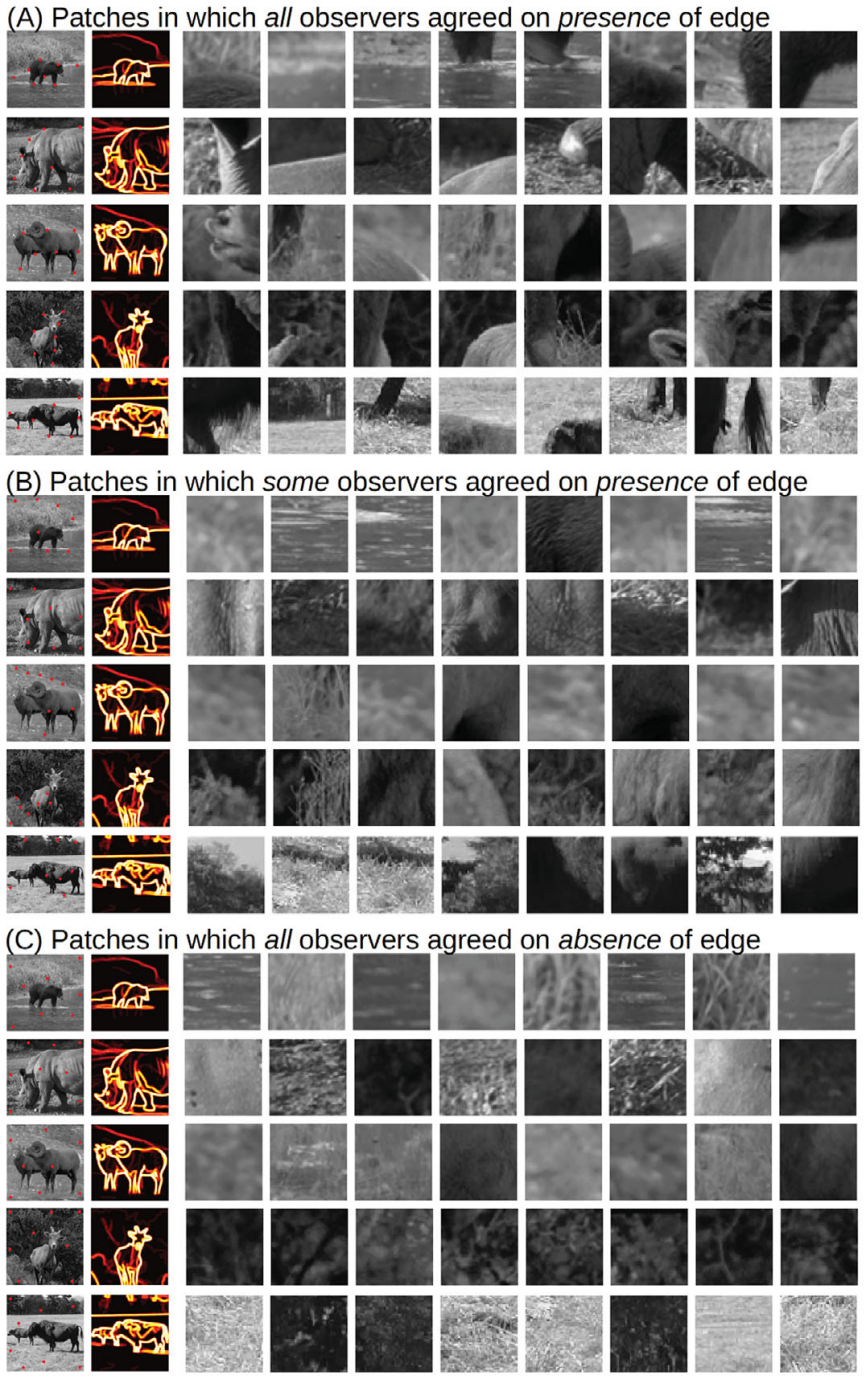
Example patches (A) with salient edges, (B) with some edges, and (C) without edges. The image on the left shows the natural image with the locations (red dots) where the eight 51×51 patches on the right were taken from. The image right next to the full image shows the summed ground-truth maps of all subjects. Salient edges were defined as edges where at least 19 observers traced an edge. (B) is defined by patches in which 5 to 15 observed detected an edge.

**Figure 9.**
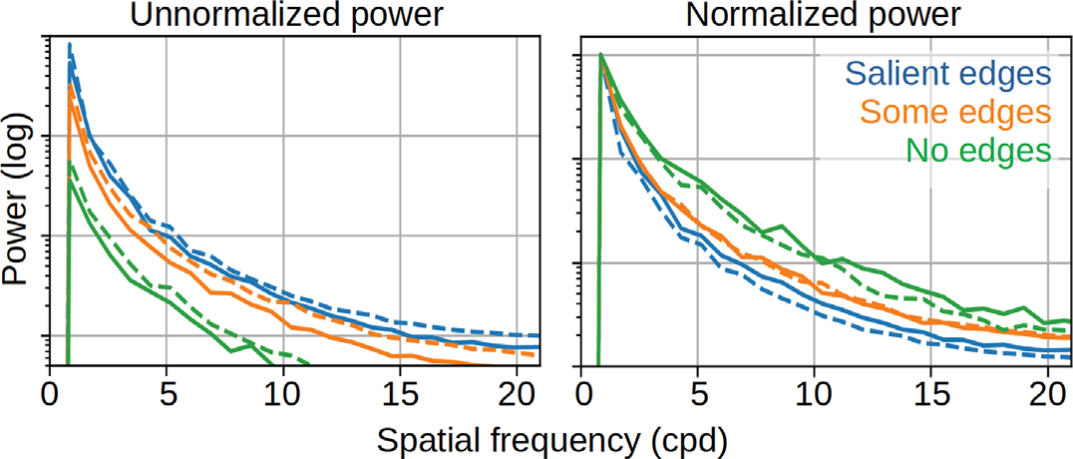
Power spectrum of the three different kinds of patches (salient, some, none) in the vertical direction (dashed lines) and horizontal direction (continuous lines). To compare relative differences between patch types, the right plot shows the normalized power (i.e. the power distributions on the left but divided by the maximal power of each graph).

A desirable characteristic of a sensitivity measure is that it is unaffected by response bias (Macmillan & Creelman, 1991). To quantify sensitivity, we compute *d-prime* (*d*′) as the difference between the *z*-transformed hit and false alarm rates:

(2) $$d^{'}=z\left(\frac{\text{Hits}}{\text{Hits}+\text{Misses}}\right)-z\left(\frac{\text{FA}}{\text{FA}+\text{CR}}\right)$$

We also compute the response bias *c* to measure whether responses of observers were biased toward either signal-present or signal-absent responses:

(3) $$\;c=-\frac{1}{2}\left[z\left(\frac{\text{Hits}}{\text{Hits}+\text{Misses}}\right)+z\left(\frac{\text{FA}}{\text{FA}+\text{CR}}\right)\right]$$

### Error margin

Pen width of our tracing tool was 1 pixel. However, observers cannot trace edges with pixel-perfect accuracy across repeated attempts. To compare the ground-truth maps with the line drawings in visual noise, we therefore apply an error margin to the ground truths (Figure 4; cf. Grigorescu et al., 2003). Misalignments within this margin are thus still counted as hits. While the choice of error margin is somewhat arbitrary, it should be large enough to accommodate small tracing inconsistencies, yet not so large that it begins to count unrelated markings as correct.

**Figure A2.**
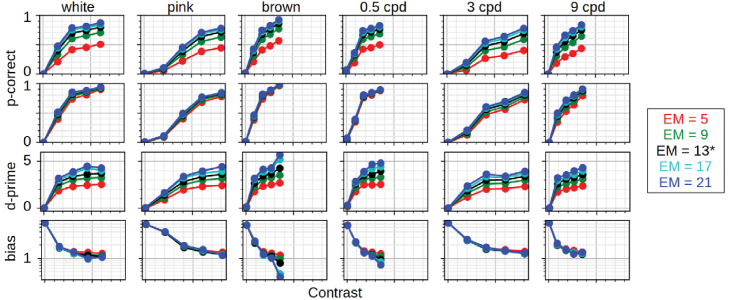
Effect of varying the error margin. From top to bottom: proportion correct, scaled proportion correct, d-prime, and response bias for different error margins. Changing the error margin mostly just scales the different curves but does not affect them qualitatively. We used an error margin of 13 pixels.

In our experiments, the exact value of the error margin had minor effects on the result patterns. It primarily scaled the performance curves up or down (see Appendix Figure A2). We therefore settled on an error margin of 2.5% of the image size (13 pixels), as it struck a reasonable balance between the two extremes illustrated in Figure 4.

### Observers

All procedures were approved by the institutional ethics committee. A total of *N* = 20 observers with normal or corrected-to-normal vision participated in the experiment. Five observers were experienced psychophysical observers, while the remaining 15 were naive to the purpose of the experiment. Each observer completed the experiment in two to three 1-hour sessions.

### Apparatus

Stimuli were created with *stimupy 1.1* (Schmittwilken, Maertens, & Vincent, 2023), and presented on a ViewPIXX 3D monitor (523 × 293 mm, 1,920 × 1,080 px, 120 Hz) using custom presentation software (available at http://github.com/computational-psychology/hrl). Monitor calibration and linearization were performed with a Minolta LS-100 photometer (Konica Minolta, Tokyo, Japan). Observers viewed the display from a fixed distance of 100 cm, maintained with a chin rest. At this distance, pixel resolution was 44 pixels per degree.

Observers used a Wacom Intuos pen tablet to draw their responses. The tablet does not feature a built-in display. Instead, its inputs were mapped in real time onto the stimulus presented on the monitor, allowing participants to draw directly over the stimulus. The drawn lines were rendered in white at a luminance of 250 cd/m^2^ superimposed in the stimulus with mean luminance of 100 cd/m^2^. Observers could correct mistakes by pressing a designated button on the tablet, which allowed them to undo traces to adjust their drawings.

The experiment was conducted under binocular viewing conditions in a dark room to minimize interference by ambient light.

## Results

### Qualitative drawing behavior

To validate our approach, we first qualitatively examined the line drawings produced by observers in the absence of noise (i.e., their individual ground truths). Figure 5 presents three example stimuli from the database, with the corresponding line drawings from all observers overlaid on top of each other.

These examples demonstrate that observers successfully performed the task, tracing lines at locations of edges. The examples also illustrate the importance of the error margin when evaluating performance, as the traces did not align at the pixel level.

Observers primarily traced edges at object boundaries, the horizon, and distinct object features. There was variability in the level of detail captured by different observers, but observers overall agreed in the locations of their traces (Figure 5). To quantify this agreement, we compared how consistent the ground-truth maps were across participants in comparison to repeated drawings within the same participant. We computed proportion correct between the ground-truth maps of all observers and compared this value to proportion correct between each observer's ground truth and their noisy maps for images with maximum contrast in each noise condition (i.e., when the image was clearest).

Proportion correct between observers was ${\bar{p}}_{between}=0.63\pm 0.14$ on average, compared to ${\bar{p}}_{within}=0.66\pm 0.13$ within observers, indicating that interobserver consistency was nearly as high as intraobserver consistency.

Observers' traces often disagreed at shadows. Some shadows caused large luminance discontinuities in the images (e.g., at the goat's neck or beneath the golf cart), but only a few observers traced the outlines of these shadows (Figure 5). We will come back to this point when we explore the features that have driven edge perception in our experiment.

### Quantitative performance measures

Figure 6A–B illustrates how proportion correct, d-prime, and response bias change with increasing image contrast across the different noise conditions. Proportion correct and d-prime increased with increasing contrast and saturated at high contrasts. The maximum proportion correct averaged across subjects did not exceed 74%. This means that even at high contrast, observers' noisy traces did not perfectly match their traces in the ground-truth maps.

We computed d-prime in addition to proportion correct, because d-prime also considers responses when the signal is absent and hence is less sensitive to response bias (Macmillan & Creelman, 1991). We found good agreement between proportion correct and d-prime (Figure 6A), which suggests that false alarm rates and correct rejections contributed little additional information about edge visibility.

**Figure A3.**
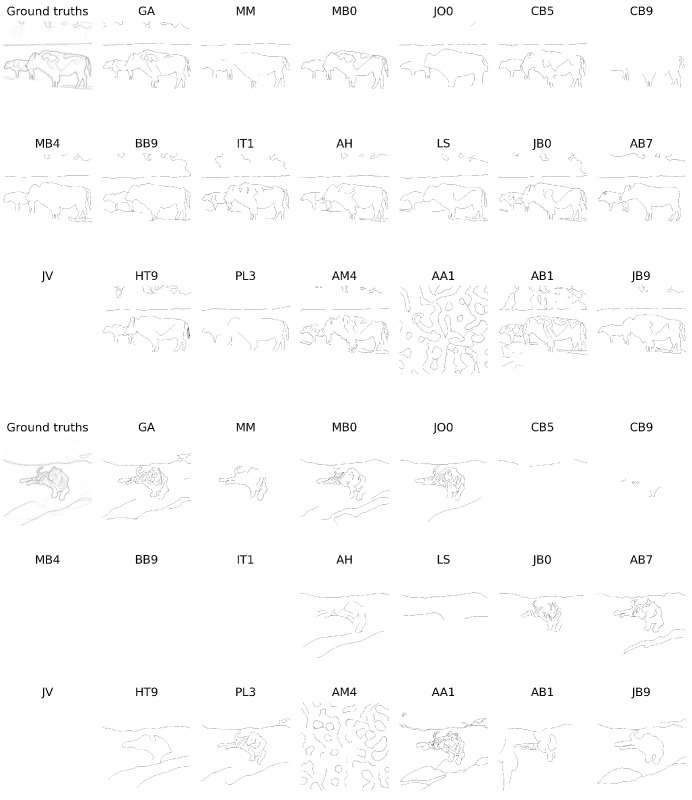
Example traces for noisy conditions of all subjects. We show the line drawings of all subjects for two example images (buffalo and tiger). Observers viewed the images at different image contrasts and masked with different kinds of noise. As a result, edges were not visible to the subjects in all cases. These examples also show that observers sometimes trace the outlines of the noise mask (AA1 and AM4). For comparison, the first image shows the stacked ground truths for all subjects (i.e., their traces in the absence of noise).

Response bias decreased as contrast increased. We observed a negative correlation between proportion correct (Figure 6A) and response bias (Figure 6B; *r* = −0.84, *p* < 1 × 10^−5^), indicating that observers traced more edges when they were barely visible, while they traced fewer edges when they were more apparent. This pattern may partially be caused by observers who traced noise because they mistook it for relevant image structures, particularly at low contrast in the presence of 0.5 cpd and white noise (see traces in Appendix Figure A3).

To quantify the relationship between edge sensitivity and image contrast, we fitted psychometric functions to observers' proportion correct data (Figure 6C). Maximum performance never reached 100%. To make the fitting of psychometric functions easier, we scaled the proportion correct measure by subjects' maximum performance. We then applied cumulative Gaussian fits to the scaled data averaged across all participants, using the *psignifit 4* software package (Schütt, Harmeling, Macke, & Wichmann, 2016). Goodness-of-fit for these psychometric functions was excellent so that we could derive a threshold equivalent, which we compared the thresholds from the previous experiment.

### Comparison to traditional approach

In a previous study (Schmittwilken et al., 2024), we measured human edge sensitivity with a more traditional approach. Using Cornsweet edges (Figure 7A, left column), observers detected edges in different types of noise in a two-alternative forced-choice (2-AFC) paradigm. We varied the spectral properties of the edges (three peak frequencies: 0.5, 3, and 9 cpd; rows in Figure 7A) and the noise masks to probe how spatial frequency selective mechanisms contribute to edge sensitivity. We observed most effective masking when noise and edge had similar spectral properties (Figure 7A, right column). Pink noise and narrowband noise centered at 3 cpd effectively masked all edges irrespective of their peak frequencies.

Here, we examined edge sensitivity in natural scenes with the same noise patterns. Figure 7B shows the sensitivity patterns obtained with natural images as a function of noise. By comparing Figures 7A and 7B, it is evident that the masking patterns are strikingly similar between the two approaches. Correlation analyses show that the pattern observed with the natural images closely aligns with the thresholds observed in the 0.5 cpd (*r* = 0.97; *p* = 0.001) and the 3 cpd edge conditions (*r* = 0.89; *p* = 0.016). The threshold pattern in the 9 cpd edge condition was clearly different from the present sensitivity pattern (*r* = 0.51; *p* = 0.292). This discrepancy is likely due to the spectral similarity between the natural images and the 0.5 cpd edge (*r* = 0.57). However, from this perspective, we might expect less agreement between the natural scenes and the 3 cpd edge, given that their stimulus spectra are minimally correlated (*r* = −0.08) and inversely correlated with the 9 cpd stimulus spectrum (*r* = −0.26).

### Features driving edge perception

To explore which image features drive edge perception in natural images, we performed an analysis inspired by Alam, Vilankar, Field, and Chandler (2014). We extracted random patches (51×51 pixels) from images where either (a) all observers traced an edge or (b) some observers (25%–75%) traced an edge, or (c) no observer traced an edge in the absence of noise. To identify these regions, we first stacked the ground-truth maps from all participants to generate the heatmaps shown in Figure 8. We then randomly selected patches whose central pixel was labeled as belonging to an edge by (a) all, (b) some, or (c) none of the observers. Patches were sampled across the entire image to avoid selecting neighboring regions. Example patches from each category are shown in Figure 8.

We make several qualitative observations when visually inspecting these patches. (a) Patches with salient edges often contain abrupt and large luminance discontinuities. These discontinuities are frequently oriented and often span the entire patch or at least a substantial portion of it. (b) Patches in which only some observers detected edges tend to show slightly weaker luminance transitions, which appear more blurred and less extensive. (c) Patches without any reported edges generally appear more homogeneous and mostly contain fine-scale textures.

One particularly interesting observation links back to the perception of shadow edges. We subjectively observed that many patches in which only some observers detected edges (25%–75% of observers) contain shadowed regions (Figure 8B). Unlike other patches with ambiguous edge detection, these shadow patches exhibit surprisingly high contrasts and span large portions of the patches. This suggests that observers tended to not trace shadow edges, even though they were similarly salient as patches with salient edges.

In addition to our visual assessment, we analyzed several quantitative features of the patches, including luminance, contrast, and spatial frequency content.

Luminance levels were found to be similar across the three categories (salient: 100.1 ± 10.8, few: 99.8 ± 13.8, none: 101.4 ± 12.7 cd/m^2^). RMS contrasts systematically increased with the perceived salience of edges (salient: 0.14 ± 0.06, few: 0.10 ± 0.04, none: 0.05 ± 0.03). This increase in contrast for salient edges supports our qualitative observation that salient edges are associated with sharper luminance transitions.

We also examined the power spectra of the patches (Figure 9). Consistent with their higher contrast, patches with salient edges exhibited greater overall power. Interestingly, more power was consistently observed in the vertical direction than in the horizontal direction, which may reflect the prevalence of horizontally oriented structures in natural scenes (Field, 1987). Even after normalizing the power spectra to account for the differences in overall power, we found distinct patterns across patch categories. Specifically, patches with salient edges showed relatively less power at high spatial frequencies compared to those without detected edges. This finding might reflect our observation that more fine-scale textural details are present in the latter.

## Discussion

Our understanding of human pattern vision rests on an experimental approach that isolates individual visual mechanisms by using well-controlled stimuli and binary decision tasks (Rust & Movshon, 2005). In the domain of human edge sensitivity, stimuli are isolated edges or gratings, and performance is measured near the detection threshold (Campbell & Robson, 1968; Shapley & Tolhurst, 1973). This approach has yielded valuable insights into the mechanisms underlying pattern vision, such as the postulate of multiple spatial frequency and orientation-selective channels (Blakemore & Campbell, 1969; Henning, Hertz, & Broadbent, 1975). It has provided the foundation for what could be called the standard of image-computable mechanistic models of early visual processing (Rust & Movshon, 2005; Graham, 2011; Schütt & Wichmann, 2017, for reviews). Here we wanted to test how these insights transfer to the rich and high-contrast inputs that are typical for our everyday visual experience (Shapley & Reid, 1985; Olshausen & Field, 2005).

There is evidence that sensitivity measured in images of natural scenes differs from sensitivity measured in simple stimuli (Bex, Solomon, & Dakin, 2009; Haun & Peli, 2013; Jarvis, Triantaphillidou, & Gupta, 2022), and that discrimination of suprathreshold contrast is not limited by the contrast sensitivity function (Georgeson & Sullivan, 1975). For edge perception context plays a crucial role: Edges are more readily perceived at object boundaries than in less salient regions of an image (Neri, 2011, Neri, 2017, Neri, 2024; Papale et al., 2024).

Yet, it is difficult to study pattern vision in real-world scenes under controlled experimental conditions, and different experimenters are willing to make different compromises between behavioral relevance and experimental control. Natural scenes are inherently rich in information, which makes isolating specific perceptual mechanisms more challenging. Moreover, what constitutes an edge in natural scenes is not evident, because gradual transitions in luminance, texture, and shadows might blur the boundaries between edges and other visual features. To address these challenges, many studies revert to simple detection or discrimination tasks, which require large amounts of data to draw reliable conclusions (Neri, 2011; Haun & Peli, 2013; Alam et al., 2014).

Here we adopt a different approach to probe human edge sensitivity in natural scenes. We asked observers to trace relevant edges in images of natural scenes in the presence and absence of two-dimensional (2D) visual noise. Edge tracing is a straightforward task that observers can naturally do. Every trial results in an edge map that provides a rich source of data. In order to measure the effect of noise on observers' ability to perceive edges in the images, we used their traces in the noise-free images as ground truths. We thus used a subjective measure of perceived edges as the standard of comparison instead of physically defined edges. This allows us to circumvent a potentially premature and somewhat arbitrary definition of what constitutes a relevant edge in a natural image. We analyzed the edge traces within a signal detection framework in order to derive a quantitative performance measure that we could compare to previous work.

### Validity of line drawings

Line drawings date back over 30,000 years (Aubert et al., 2014; Hoffmann et al., 2018). Their simplicity and effectiveness in conveying complex visual information have long fascinated artists and scientists alike (Fan et al., 2023). Line drawings are universally recognized across cultures, developmental stages, and species, even by infants (Yonas & Arterberry, 1994), nonhuman primates (Tanaka, 2007), and communities without exposure to pictorial art (Kennedy & Ross, 1975). This widespread recognition is noteworthy, because natural scenes lack distinct visual features that resemble lines (Sayim & Cavanagh, 2011). It has been suggested that line drawings stimulate similar visual representations as natural scenes, and we therefore accept them as a decent surrogate (Ishai, Ungerleider, Martin, & Haxby, 2000; Walther, Chai, Caddigan, Beck, & Fei-Fei, 2011). This makes them a powerful tool to study perceptual mechanisms in a more naturalistic setting.

Line drawings also provide information about individual differences, which are not available in a simple button press (Green & Swets, 1966). The drawn edges reflect how observers prioritize and perceptually organize the visual input (Sheng, Wilder, & Walther, 2021; Fan et al., 2023). They might also reflect decision processes (e.g., which lines to draw and what level of detail to provide), which would be cognitive rather than perceptual processes. Parts of observers' edge maps and of the interobserver variability might thus reflect processes that are not directly related to perception and potentially concern the validity of the task (Bainbridge, 2022). We would argue that such processes also play a role when observers press a button. Here at least we make part of this explicit and openly visible, and these considerations might be addressed in future experiments. For example, we might record how the traces evolve over time. This allows to compare variability in those traces that observers draw first and those that they draw last. The lines that are drawn first might be less affected by considerations about how much effort to invest in the drawing.

### Noise as an experimental tool

A key component in our approach to explore edge sensitivity with line drawings is visual noise. Visual noise is a powerful tool in visual psychophysics. It is broadly applicable and has been used in a wide range of visual tasks, including letter and face recognition, attention, and perceptual learning (Allard, Faubert, & Pelli, 2015, for review). Uncorrelated, broadband noise is commonly used in a so-called ideal observer analysis. The idea is to use different types of external noises to differentiate different types of internal noise and to establish benchmarks for visual performance (Legge, Kersten, & Burgess, 1987; Pelli & Farell, 1999). Correlated, bandpass-filtered noise can be used to probe the putative spatial frequency selective mechanisms that govern visual perception (Stromeyer, Lange, & Ganz, 1974; Solomon & Pelli, 1994; Betz, Shapley, Wichmann, & Maertens, 2015). It also allows to probe the adaptability of visual strategies (or channels) and interactions between spatial frequency selective channels (Kersten, 1987; Conrey & Gold, 2009). Here we have used noise to exploit these functions (i.e., to test for channel-specific interference effects). Another advantage of noise is that it allows us to test sensitivity for images with a clearly supra-threshold contrast (i.e., under viewing conditions that more closely mirror real-world vision). We tested the same noise types as in a previous experiment with simple edge stimuli.

To quantify the edge traces in different noise conditions, we defined a performance measure that is rooted in SDT (Green & Swets, 1966). This allows us to separate sensitivity (i.e., the ability to discriminate signals from noise) from decision processes, which might be subject to internal or external bias. To apply this framework, we treat individual pixels of each edge map as discrete trials and categorize pixels that are traced into hits, misses, false alarms, and correct rejections (Figure 3). We derive edge-tracing functions analogous to psychometric curves. Although this approach violates the SDT assumption of trial independence, it offers a principled way to link behavioral variability to stimulus properties, and to directly compare the results with traditional threshold data.

We observed that edge sensitivity in the present experiment was highest for intermediate spatial frequencies between one and five cycles per degree. This is consistent with our previous results (Schmittwilken et al., 2024) and with the peak of the contrast sensitivity function (Campbell & Robson, 1968; Shapley & Tolhurst, 1973). We further observed similar interference patterns for edge sensitivity in natural stimuli and the 0.5 cpd as well as the 3 cpd Cornsweet edges (Figure 7). The similarity to the 0.5 cpd edge is expected, because its spectrum is similar to that of natural scenes. The resemblance to the 3 cpd edge is less intuitive and cannot be attributed to similarity of the respective power spectra.

### Image features related to edge perception

We also analyzed which image features contribute to edge perception. Observers were more likely to trace edges in regions of higher contrast than in regions of lower contrast. There was no difference in the number of traced edges between patches of different mean luminance (Alam et al., 2014). Observers consistently traced edges near object boundaries, which is intuitively plausible and consistent with previous work (Neri, 2017, Neri, 2024; Papale et al., 2024). We also observed that not all salient (i.e., high contrast) edges were traced, in particular when those edges demarcated shadow boundaries (Figure 8). On the other hand, observers traced edges of “good continuation,” such as object boundaries, even when they were of very low image contrast and even though we explicitly instructed subjects to not trace them. (Neri, 2024, Neri, 2017) recently measured contrast sensitivity in a reverse correlation paradigm at image- or object-defined boundaries, either in a virtual (3D) black-and-white world or in images (2D) of natural scenes. He observed higher sensitivity at object- compared to image-based boundaries in natural images (Neri, 2017). In the virtual reality (VR), simplified black-and-white world, sensitivity at object-based boundaries was at best as good as that at image-based boundaries (Neri, 2024). The visual system is obviously very quick to establish representations of object-based boundaries. However, it remains an open question about what are the decisive cues in the visual input that trigger object segmentation. Insights gained with line drawings about where observers perceive boundaries might help to identify conditions in which image- and perception-based boundaries divert. These conditions can then be used to isolate the mechanisms involved in building these representations by quantitatively studying sensitivity in paradigms such as reverse correlation.

Our approach points to some new research avenues. The exploratory analysis of perception-based image patches yielded interesting patterns that deserve more systematic follow-up. One question concerns the perception of shadows: To what extent are participants sensitive to shadows and their boundaries, and how do they distinguish them from material or object edges (see also Sayim & Cavanagh, 2011)? We also noted an apparent overrepresentation of horizontal and vertical edges: Does this reflect a perceptual bias, a drawing bias, the statistical structure of natural scenes, or any combination of those? The edge traces also provide a rich source of data to study interindividual differences. In the current design, each participant viewed a unique set of images. Future studies could present identical stimuli across observers to better characterize individual strategies. Such data could reveal individual perceptual biases and provide information on how different observers weight different visual cues for edge tracing.

We think that edge tracing and related drawing methods deserve to be employed more to study vision in the context for which it evolved: “messy” (as opposed to experimentally controlled) but structured real-world scenes. While the control of the reductionist approach remains invaluable (Rust & Movshon, 2005), it should be complemented by methods that probe perception under more natural conditions (Olshausen & Field, 2005; Bex & Langley, 2007). Line drawings provide a step in this direction. Here we combined natural stimuli with noise masking and summarized the edge traces in parameters of signal detection theory. This was our attempt to bridge the gap between ecological validity and psychophysical rigor, and it gave us the flexibility to study visual perception in scenes that resemble those we encounter in daily life. We think our approach can become a useful tool in vision science, and it might also provide benchmark data for testing models of both human and machine perception.

To conclude, we demonstrate that edge sensitivity in natural scenes can be measured effectively using line drawings. Our approach replicates findings obtained with more traditional psychophysical measures, yielding additional observations that might depend on specific image features and/or the context of natural scenes. It offers a rich dataset for both theoretical and applied investigation. Our approach expands the toolbox with which we study pattern vision, by incorporating more natural stimuli and more natural responses, while keeping the rigor of data analysis.

## Footnotes

1 Our performance quantification and terminology are largely inspired by similar approaches in computer vision to quantify the performance of computer vision algorithms against human-labeled contours maps (Grigorescu et al., 2003).
